# Bile Acids and Short-Chain Fatty Acids Are Modulated after Onion and Apple Consumption in Obese Zucker Rats

**DOI:** 10.3390/nu15133035

**Published:** 2023-07-05

**Authors:** Claudia Balderas, Begoña de Ancos, Concepción Sánchez-Moreno

**Affiliations:** Institute of Food Science, Technology and Nutrition (ICTAN), Spanish National Research Council (CSIC), ES-28040 Madrid, Spainancos@ictan.csic.es (B.d.A.)

**Keywords:** functional foods and ingredients, prebiotic effect, microbiome, short-chain fatty acids, primary and secondary bile acids, conjugated bile acids, glycine- and taurine-conjugated bile acids

## Abstract

Gut microorganisms are involved in the development and severity of different cardiovascular diseases, and increasing evidence has indicated that dietary fibre and polyphenols can interact with the intestinal microbiota. The study objective was to investigate the effect of onion and apple intake on the major types of microbial-derived molecules, such as short-chain fatty acids (SCFAs) and bile acids (BAs). Obese Zucker rats were randomly assigned (*n* = eight rats/group) to a standard diet (OC), a standard diet/10% onion (OO), or a standard diet/10% apple (OA). Lean Zucker rats fed a standard diet served as a lean control (LC) group. Faecal samples were collected at baseline, and 8 weeks later, the composition of the microbial community was measured, and BA and SCFA levels were determined using high-performance liquid chromatography–mass spectrometry (HPLC-MS) and gas chromatography–mass spectrometry (GC-MS), respectively. Rats fed onion- and apple-enriched diets had increased abundance of beneficial bacteria, such as *Bifidobacterium* spp. and *Lactobacillus* spp., enhanced SCFAs (acetic, propionic, isobutyric, and valeric acids), decreased excretion of some BAs, mainly of the primary (CA, α-MCA, and β-MCA) and secondary type (ω-MCA, HDCA, NCA, DCA, and LCA), and increased amount of taurine- and glycine-conjugated BAs compared to the OC group. The contribution of specific bioactive compounds and their metabolites in the regulation of the microbiome and the pathways linked to SCFA and BA formation and their relationship with some diseases needs further research.

## 1. Introduction

Bile acids (BAs) play a critical role in maintaining overall health, as they are essential endogenous compounds synthesised from cholesterol in the liver and metabolised in the intestine by the gut microbiota. The circulation of BAs is very efficient and plays an important physiological role in lipid absorption and the regulation of cholesterol homeostasis [1,2]. Humans and rodents exhibit substantial differences in respect of bile acid composition. The BAs that are newly synthesised from cholesterol and formed in the hepatocytes are termed primary BAs. Cholesterol is converted into BAs by pathways that involve 17 different enzymes, many of which are preferentially expressed in the liver [3]. Under normal physiological conditions, the primary BAs are synthesised via two major pathways, the classical (neutral) and the alternative (acidic) pathways [4]. The primary BAs produced in humans are cholic acid (CA) and chenodeoxycholic acid (CDCA), while rodents produce CA, CDCA, α-muricholic acid (α-MCA), and β-muricholic acid (β-MCA). After biosynthesis from cholesterol and before excretion from the hepatocyte, bile acid molecules are conjugated with glycine or taurine, which converts a weak acid to a strong acid. In contrast, the BAs generated by subsequent modifications carried out by the gut microbiota are termed secondary BAs, including deoxycholic acid (DCA), lithocholic acid (LCA), ursodeoxycholic acid (UDCA), ω-muricholic acid (ω-MCA), and their conjugated counterparts [4,5,6]. Secondary BAs are hypothesised to be interkingdom signalling molecules. By understanding the dynamic intricacies in the gut microbiota–bile acid–host axis, insight into a variety of disease states could be elucidated [7]. Obesity is associated with an altered BA metabolism, and these products of microbial metabolism, along with short-chain fatty acids (SCFAs), among others, have been associated with an increased risk of cardiovascular disease, inflammation, and metabolic disorders [8,9]. The relationship between the BAs and microbiota appears to be bidirectional; gut microbes influence BA composition, and, in turn, BAs modulate the gut microbial community [10,11]. An imbalance in gut microbiota is considered to be an important factor in chronic inflammation diseases, such as obesity and autoimmune and metabolic diseases [12,13]. Most diseases only lead to an increase or decrease in the concentration of one or several BAs but not total BAs. The separation of various BAs and the detection of their concentration changes in samples are conducive to scientific research.

Diet, especially fibre intake, serves as a potent modulator of the gut microbiome [14]. Various studies have shown that different types of dietary fibres or fibres with different physiological and chemical properties are related to changes in the bile acid profile of rats [15]. Higher fibre diets are associated with various health benefits, in part due to their ability to increase microbial diversity and promote the growth of beneficial intestinal bacteria, such as *Bifidobacteria*, *Lactobacillus*, and *Eubacterium*, which produce SCFAs [16].

SCFAs are bioactive metabolites excreted by anaerobic bacteria in the colon after the degradation of dietary fibre and indigestible carbohydrates. The SCFA concentration in the gut depends on the microbiota composition, gut transit time, the regulation of host energy metabolism, and the fibre content of the host diet. Acetate, propionate, and butyrate are the most abundant, representing 90–95% of the total SCFAs present in the colon [17]. Dietary fibre and its associated compounds digested by the microbiota and their resulting metabolites, especially SCFAs, are significantly associated with health beneficial effects, such as via proposed anti-inflammatory mechanisms [18]. Thus, microbial metabolites including SCFAs may be suitable biomarkers in clinical practice, as their detection in blood plasma and in faeces is usually affordable. Emerging evidence based on numerous animal studies has shown that gut microbiota and its metabolites, particularly SCFAs, play an important role in hypertension, inflammatory bowel disease, diabetes, and obesity, among other diseases [19,20,21,22,23,24,25].

Onions and apples are both considered sources of dietary fibre and other phytochemicals, such as polyphenols, although their fibre content and compositions may differ. Onions are rich mainly in flavonoids, highlighting the presence of quercetin derivatives, such as quercetin 3,4′-*O*-diglucoside and quercetin 4′-*O*-glucoside, in addition to other glycosides of quercetin, kaempferol and isorhamnetin, and anthocyanins [26]. In apples, quercetin (mainly in the skin) is found as a mixture of the derivatives of 3-*O*-galactoside, 3-*O*-glucoside, 3-*O*-rhamnoside, and 3-*O*-rutinoside, in addition to other phenolic compounds, such as the derivatives of hydroxycinnamic acid (chlorogenic acid and caffeic acid) and flavanols, such as (+)-catechin, (−)-epicatechin, and the chalcones phloridzin and phloretin [27]. Animal studies suggest that polyphenol or polyphenol rich foods induce changes within gut microbiota and its metabolic output of trimethylamine *N*-oxide, SCFAs, BAs, and small phenolic acids [28]. Therefore, including these plant-based foods in the diet can provide essential nutrients and contribute to overall gut health. Onions, particularly the outer layers, are rich sources of both soluble and insoluble fibres. Soluble fibre, in the form of fructooligosaccharides (FOSs) and inulin, where the FOSs are one of the most acceptable prebiotic substances together with galactooligosacharides and lactulose [29], while insoluble fibre, such as cellulose and hemicellulose, provide bulk to the stool and help maintain regular bowel movements [30]. Apples, on other hand, are known for their high content of soluble fibre, specifically pectin. Pectin is a complex polysaccharide that has prebiotic properties, selectively stimulating the growth of health-promoting gut bacteria [31]. The fermentation of pectin by gut microbiota results in the production of SCFAs. Additionally, pectin has been shown to bind to BAs in the intestine, reducing their reabsorptions and promoting the synthesis of new BAs from cholesterol, which can help lower circulating cholesterol levels [31,32].

Previously, it has been shown that oxidative stress biomarkers (protein carbonyls, 8-hydroxy-20-deoxyguanosine, and 8-epi-prostaglandin F_2α_), inflammatory and vascular injury biomarkers (PAI-1, TIMP-1, VEGF, sICAM-1, sE-Selectin, and MCP-1), and leptin were lower in rats fed the onion and apple diets compared with rats fed the standard diet. In addition, endothelial impairment was partially reversed, and the superoxide content and gene expression of NLRP3, NFKβ1, and COX2 decreased [33]. González-Peña et al. (2017) [34] reported significant changes in specific primary and secondary BAs in both the unconjugated and conjugated forms in rats fed a high-cholesterol diet enriched with onions. In addition, Fernández-Jalao et al. (2021) [35] reported phenolic metabolite and SCFA production as well as the modulation of certain bacterial groups, exerted by an onion ingredient treated using high pressure processing and subjected to a dynamic gastrointestinal digestion and colon fermentation simulator. Based on these results and the extended literature findings regarding the relationships among dietary patterns, gut microbiota, BA and SCFA metabolism, and metabolic health, the purpose of the present study was to investigate the effect of the onion and apple intake in an obesity animal model on the major types of microbial-derived molecules, such as BAs and SCFAs, linked to the development of inflammation and metabolic disturbances to understand the interactions between diet, gut microbiota, and BA and SCFA metabolism.

## 2. Materials and Methods

### 2.1. Onion and Apple Powder Preparation

Onion and apple powder preparation is displayed in Supplementary Materials and Methods. Supplementary Table S1 shows the nutritional composition, phytochemical compounds, and antioxidant activity of the onion and apple powders. Analyses were carried out using the methods previously cited in Balderas et al. (2022) [33].

### 2.2. Animal Model and Experimental Design

The present study was approved by the Spanish Ministry of Economy, Industry, and Competitiveness Advisory Committee (project AGL2016-76817-R) and by the Ethics Committee of the Complutense University of Madrid (Spain) (reference: PROEX 133/16). All experiments were performed in compliance with Directive 2010/63/UE regarding the protection of animals used for scientific purposes. All necessary steps were taken to prevent any potential animal suffering. Animal model and experimental design are displayed in Supplementary Materials and Methods [36,37,38,39,40]. Supplementary Table S2 shows the composition of the experimental diets [33].

### 2.3. Faeces Collection and Caecum Sampling

Once a week, the animals were housed in individual metabolic cages for 24 h for faecal sample collection. Faeces were stored at −20 °C until analysis. For the microbial analysis and SCFA and BA determination, the faeces corresponding to Week 0 (start or baseline) and Week 8 (end of the experimental feeding trial) were used. At Week 8 of the feeding trial, in order to avoid interassay variations that could affect the comparison of data from the different groups, animals in fasting conditions were euthanised by decapitation, taking randomly one animal at a time, from each one of four groups. The caecum was collected and weighed before being frozen in liquid nitrogen and stored at −80 °C until analysis.

### 2.4. Caecum pH and Antioxidant Activity Determination

A portion of the caecal (0.5 g) content was diluted in water immediately after sampling; the pH was measured using a microelectrode (Crison micropH2000). 2,20-azinobis(3-ethylbenzothiazoline-6-sulfonic acid) radical cation (ABTS^•+^) scavenging capacity and ferric-reducing antioxidant power (FRAP) in caecum were measured using the methods described in Colina-Coca et al. (2017) [38].

### 2.5. Microbial Analysis

A sample of approximately 1 g of faeces was diluted in 9 mL peptone water (Cultimed-Panreac, San Fernando de Henares, Madrid, Spain) and homogenised with vortex (Vortex model LabDancer S40, VWR; Radnor, PA, USA); in addition, a series of 10-fold dilution (10^−1^–10^−11^) was prepared. A given amount of each dilution (1 mL) was plated onto a selective media. The methods for the determination of total aerobic bacteria count, *Enterobacter* spp., β-glucuronidase positive *Escherichia coli*, sulphite-reducing *Clostridium* spp., *Bifidobacterium* spp., and *Lactobacillus* spp. are described in Colina-Coca et al. (2017) [38]. Colony-forming units (CFUs) were defined as colonies measuring at least 1 mm in diameter. Microbial counts were expressed as log CFU/g fresh weight (fw).

### 2.6. Short-Chain Fatty Acid Analysis

#### 2.6.1. Short-Chain Fatty Acid Extraction

Faecal samples were weighed and suspended in 1 mL of 0.5% phosphoric acid and frozen at −20 °C immediately after collection. Once thawed, the faecal suspensions were homogenised with a vortex for about 2 min and centrifuged for 10 min at 13,951× *g*. Supernatants were extracted with 1 mL of butanol and centrifuged for 10 min at 13,951× *g*. Prior to analysis, a 200 µL volume of the organic phase was transferred into a tube, and 100 µL of 4-methyl valeric acid was added as internal standard (394 µM final concentration). Samples were directly injected onto the GC-MS column.

#### 2.6.2. Short-Chain Fatty Acid Identification and Quantification Using Gas Chromatography–Mass Spectrometry (GC–MS)

The GC-MS system consisted of an Agilent 7890A gas chromatograph (Agilent Technologies, Palo Alto, CA, USA) equipped with an automatic injector G2613A Series (Agilent) and coupled with a mass spectrometer (5975C). Column DB-WAXtr (60 m × 0.25 mm × 0.25 µm) and helium as carried gas (1.5 mL/min) were used for the separation. Samples (2 µL) were injected in splitless mode with an injector temperature of 250 °C. The column temperature was initially 50 °C; after 2 min, it was gradually increased 15 °C/min until 150 °C, then increased 5 °C/min until 200 °C. To reach the final temperature of 240 °C, it was increased 15 °C/min. The final temperature was maintained for 20 min (total run time 41.3 min).

Solvent delay was 3.5 min. The detector was operated in electron impact ionization mode (electron energy 70 eV), scanning the 30–250 *m*/*z* range. The temperature of the ion source, quadrupole, and interface were 230, 150, and 280 °C, respectively. All samples were analysed in randomised order.

The concentration (µM) of each SCFA was calculated using the linear regression equations (*R*^2^ ≥ 0.99) from the corresponding curves of standards obtained with six different concentrations. A characteristic single ion was selected for the quantification of each compound. Standards employed were acetic, propionic, isobutyric, butyric, isovaleric, valeric, and caproic acids (Sigma Chemical Co., St. Louis, MO, USA), employing the Enhanced ChemStation G1701DA software and the Nist 08 and HP-Wiley 138 libraries. Results were expressed as µmol/g fresh weight (fw).

### 2.7. Bile Acid Analysis

#### 2.7.1. Bile Acid Extraction

A total of 100 mg of each pool sample was weighed into a Pyrex tube, and 1.5 mL of ethanol was added and mixed using vortex vigorously for 1 min. The samples were subjected to sonication for 2 min and then heated at 80 °C for 30 min. After cooling down to room temperature, the samples were heated at 100 °C for 3 min and centrifuged at 18,894× *g* for 10 min at 4 °C. The supernatants were carefully transferred to the new tubes with 500 µL of ethanol, followed by centrifugation at 18,894× *g* for 10 min at 4 °C. The collected supernatants were dried by evaporation in a SpeedVac concentrator. The dried extracts were resuspended in one millilitre of methanol and transferred into HPLC vials after being filtered through a 0.22 μm pore size filter.

#### 2.7.2. Bile Acid Identification Using High-Performance Liquid Chromatography–Quadrupole Time-of-Flight Mass Spectrometry (HPLC–QTOF MS)

The chromatography was performed on an Agilent 1200 series HPLC (Agilent Technologies, Waldbronn, Germany) using a C18 Hypersil ODS stainless steel column (250 mm × 4.6 mm, 5 μm) (Teknokroma, Barcelona, Spain) at 60 °C. Mobile phases consisted of 5 mM ammonium acetate in Milli Q-water (A) and methanol (B). Reverse phase was employed for the separation using gradient elution mode, and the separation was carried out in 30 min under the following conditions: 8 min 65% B; 20 min 95% B; 25 min 5% B. The column was re-equilibrated to starting conditions for 5 min prior to each analysis. Flow rate was 0.8 mL/min, and injection volume was 3 µL.

The system was coupled to hybrid quadrupole–time of flight mass spectrometer with JetStream ESI source QTOF-MS (G6530, Agilent Technologies, Waldbronn, Germany), operating in negative ionization mode, following mass spectrometer parameters: capillary voltage set at −3500 V, fragmentor voltage was 200 V, and skimmer voltage was 65 V. The drying gas temperature was set to 300 °C, the sheath gas temperature was set to 250 °C, nebuliser gas flow rate was set to 12 L/min, and the nebuliser pressure was set to 45 psig. Data acquisition was collected in centroid mode using a scan rate of 1.01 scan per second, operated in full scan mode from 50 to 1200 *m*/*z*. During the analysis, two reference masses, 121.0508 *m*/*z* and 922.0097 *m*/*z*, were continuously measured. All samples were analysed in randomised order.

MS/MS collision energy was set at 10, 20, and 40 V. Data acquisition and MS and MS/MS data were processed through MassHunter Workstation software (version B.08.00, Agilent Technologies, Waldbronn, Germany).

The raw data files from LC-QTOF-MS were visually inspected and processed using the molecular feature extraction (MFE) tool in the MassHunter™ Qualitative Analysis software package, version B05.00 (Agilent Technologies Inc., Santa Clara, CA, USA). The MFE algorithm groups ions related by charge state, isotopic distribution, and/or the presence of adducts and dimers using the accuracy of the mass measurements.

### 2.8. Statistical Analysis

The univariate statistical analysis was performed using IBM SPSS Statistics Version 28.0 (IBM Corp., Armonk, NY, USA). The results were expressed as mean ± standard deviation (SD). Data were analysed using one-way ANOVA. In order to verify the homogeneity of the variances, a Levene’s test was applied. Tamhane’s T2 (equal variances not assumed) or Bonferroni (equal variances assumed) post hoc tests were used to determine differences between groups (*p* < 0.05). Mean values within the same group (LC, OC, OO, OA) at the start (baseline or Week 0) and the end of the experimental trial (Week 8) for some parameters were statistically tested using Student’s *t*-test (*p* < 0.05).

The multivariate statistical analysis was performed using Unscrambler 11.0 and MetaboAnalyst 5.0 [41] software. In order to evaluate the distribution of samples and identify the variance between the groups of study, classification using unsupervised principal component analysis (PCA), supervised analysis based on partial least-squares discriminant analysis (PLS-DA), the values of variable importance in the projection (VIP), and heatmaps were performed.

## 3. Results

### 3.1. Prebiotic Effect

Within the obese groups, there was not a significant change in the body weight gain. The food efficiency ratio was significantly lower in the OO and OA groups compared with the OC group, which indicates the positive effect of dietary interventions [33]. There were no statistically significant differences when analysing the caecum pH and dry and fresh weight (Table 1).

At the beginning of the experiment, the pH of the faeces also did not show any significant differences among the four groups. However, the OO (7.35 ± 0.43) and OA (7.07 ± 0.37) groups showed a significant increased faecal pH at the end of experiment compared to the OC group (6.73 ± 0.28).

The antioxidant capacity using the ABTS method in the caecum was higher in the LC group (3.16 ± 0.06 μmol TE/100 g fw) and significantly increased after onion (OO: 3.23 ± 0.13 μmol TE/100 g fw) and apple consumption (OA: 3.00 ± 0.17 μmol TE/100 g fw) compared with the OC group (2.45 ± 0.15 μmol TE/100 g fw). The results reflected the same trend when the antioxidant capacity was measured using the FRAP method (Table 1).

The abundance of gut microbiota was analysed in faeces in order to investigate the changes in microbial species in response to diet. The results in all groups showed that the total aerobic bacteria population decreased at the end of the 8-week period. However, when the groups were analysed individually after 8 weeks of experimental feeding, the total aerobic bacteria count was significantly higher in the OC group (8.79 ± 0.02 log CFU/g fw) compared to the OO (8.12 ± 0.06 log CFU/g fw), OA (7.88 ± 0.28 log CFU/g fw), and LC (6.91 ± 0.42 log CFU/g fw) groups. A similar trend was shown in *Enterobacter* spp. after the 8-week experimental period, with the OC group (8.06 ± 0.47 log CFU/g fw) compared to OO (7.84 ± 0.13 log CFU/g fw), OA (7.20 ± 0.60 log CFU/g fw), and LC (7.48 ± 0.16 log CFU/g fw) groups. Within the obese groups, at the end of the 8-week period, the levels of *Escherichia coli* were significantly higher in the onion- (OO: 8.44 ± 0.18 log CFU/g fw) and apple- (OA: 8.61 ± 0.15 log CFU/g fw) supplemented groups compared with the OC group (7.99 ± 0.47 log CFU/g fw). At Week 0, *Clostridium* spp. in the OC, OO, and OA groups (4.57 ± 0.39, 4.55 ± 0.54, 4.65 ± 0.53 log CFU/g fw, respectively) were significantly lower than in the LC group (6.64 ± 0.14 log CFU/g fw). At Week 8, *Clostridium* spp. increased in the obese groups (OC: 6.61 ± 0.05 log CFU/g fw, OO: 6.42 ± 0.09 log CFU/g fw, OA: 6.57 ± 0.33 log CFU/g fw), although no significant differences among the groups were observed. *Bifidobacterium* spp. and *Lactobacillus* spp. at Week 0 showed significantly higher levels in the LC group (9.09 ± 0.03 and 9.07 ± 0.09 log CFU/g fw, respectively) compared with the OC (8.49 ± 0.13 and 8.58 ± 0.13 log CFU/g fw, respectively), OO (8.50 ± 0.40 and 8.53 ± 0.24 log CFU/g fw, respectively), and OA (8.51 ± 0.35 and 8.55 ± 0.06 log CFU/g fw, respectively) groups. After 8 weeks of the experimental diet, *Bifidobacterium* spp. and *Lactobacillus* spp. followed the same trend as at the beginning of the experiment in the LC group (8.32 ± 0.11 and 8.35 ± 0.10 log CFU/g fw, respectively) compared with the OC group (7.88 ± 0.04 and 7.67 ± 0.02 log CFU/g fw, respectively). On the other hand, in general, the groups supplemented with onion and apple showed a higher concentration of *Bifidobacterium* spp. and *Lactobacillus* spp. (OO: 7.77 ± 0.14 and 7.85 ± 0.08 log CFU/g fw, respectively) (OA: 7.99 ± 0.03 and 8.11 ± 0.02 log CFU/g fw, respectively) compared with the OC group.

### 3.2. Faecal Short-Chain Fatty Acid Composition in Response to Dietary Onion and Apple Intake

The faecal SCFA contents are shown in Table 2. The two major SFCAs were acetic and propionic acids.

After 8 weeks of experimental feeding, the LC group showed greater concentration in most of the SFCAs in comparison to the OC group, particularly acetic (46.32 ± 4.54 vs. 35.58 ± 1.45 µmol/g fw), propionic (5.47 ± 0.75 vs. 2.61 ± 0.03 µmol/g fw), isobutyric (0.46 ± 0.06 vs. 0.32 ± 0.01 µmol/g fw), butyric (0.27 ± 0.02 vs. 0.13 ± 0.02 µmol/g fw), and isovaleric (0.31 ± 0.05 vs. 0.19 ± 0.05 µmol/g fw) acids. There was no significant difference in caproic acid between the LC (0.13 ± 0.01 µmol/g fw) and OC (0.12 ± 0.004 µmol/g fw) groups, while valeric acid was lower in the LC group (0.11 ± 0.01 µmol/g fw) compared with the OC group (0.15 ± 0.02 µmol/g fw).

Onion and apple supplementation changed the concentration of SCFAs: acetic (55.01 ± 4.98 and 45.42 ± 3.92 µmol/g fw, respectively), propionic (4.40 ± 0.17 and 4.42 ± 1.02 µmol/g fw, respectively), isobutyric (0.39 ± 0.04 and 0.35 ± 0.02 µmol/g fw, respectively), and valeric (0.24 ± 0.01 and 0.37 ± 0.02 µmol/g fw, respectively) acids were significantly higher compared to those in the OC group. There were no significant differences in the caproic acid concentration between the OO (0.12 ± 0.01 µmol/g fw) and OA (0.11 ± 0.01 µmol/g fw) groups with regard to the OC group (0.12 ± 0.004 µmol/g fw).

### 3.3. Bile Acid Content in Response to Dietary Onion and Apple Intake

The chromatograms for the analysis were obtained from 32 samples at 0 and 8 weeks of the experimental period and 14 quality controls (QCs). All chromatograms were aligned, and the data were filtered to remove the noise. The samples were categorised as coming from lean or obese at Week 0 and LC, OO, OC, and OA at Week 8. The robustness of the analytical procedure was evaluated using the principal component analysis (PCA) method and was proved by the clustering of QC samples. To evaluate the bile acid level differences between the groups, the data were processed with the QCs removed from the data matrix.

In total, 22 BAs were identified using the LC-MS negative mode and further evaluated. The results are shown in Table 3, with their identification confirmed through their characteristic fragments provided by the databases and the literature and by comparison with the MS/MS spectrum of the commercial analytical standard when it was available.

Regarding the multivariate data analysis and to discriminate between the groups, principal component analysis (PCA) and partial least-squares discriminant analysis (PLS-DA) models were built, using log transformation and autoscaling (mean-centered and divided by the standard deviation of each variable). The ellipse in all PCA score plots is the 95% confidence interval for each model.

**Figure 1:**
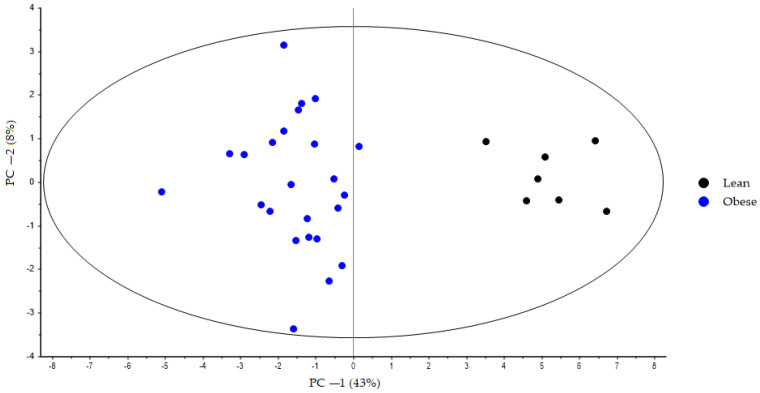
PCA score plot based on bile acid faecal samples at the start of the experimental trial (Week 0) from lean (black dots) and obese (blue dots) groups generated using Unscrambler 11.0 software. All samples were analysed in negative ionization mode. Both groups were fed a standard diet during the adaptation period. PCA, principal component analysis.

During the first step, the bile acid profiles of the lean and obese groups at Week 0 were compared using PCA. For all animals, the first principal component (PC-1) explains 43% of the variance. The lean group is markedly separated from the obese group along PC-1 (Figure 1).

**Figure 2:**
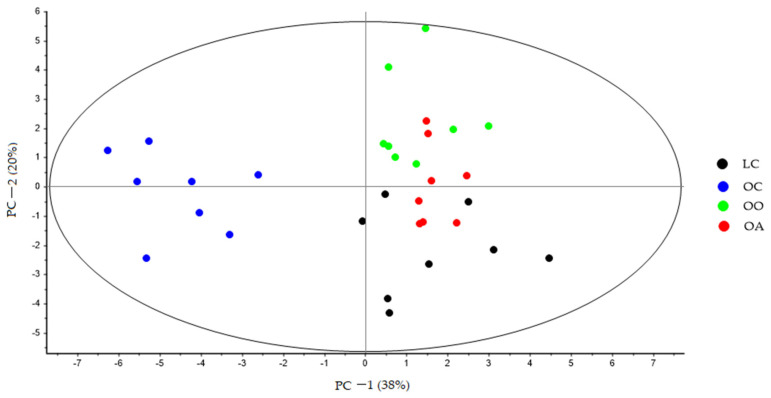
PCA score plot of bile acid faecal samples at the end of the experimental trial (Week 8) from LC (black dots), OC (blue dots), OO (green dots), and OA (red dots) groups generated using Unscrambler 11.0 software. LC, lean control: lean Zucker rats fed a standard diet; OC, obese control: obese Zucker rats fed a standard diet; OO, obese onion 10%: obese Zucker rats fed a standard diet containing 10% onion; OA, obese apple 10%: obese Zucker rats fed a standard diet containing 10% apple. PCA, principal component analysis.

**Figure 3:**
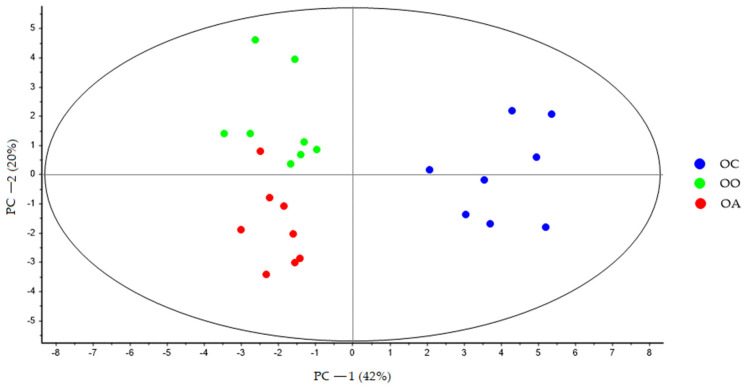
PCA score plot of bile acid faecal samples at the end of the experimental trial (Week 8) from OC (blue dots), OO (green dots), and OA (red dots) groups generated using Unscrambler 11.0 software. OC, obese control: obese Zucker rats fed a standard diet; OO, obese onion 10%: obese Zucker rats fed a standard diet containing 10% onion; OA, obese apple 10%: obese Zucker rats fed a standard diet containing 10% apple. PCA, principal component analysis.

At the end of the experiment, the PCA showed a clear separation between all the groups, and PC-1 explains 38% of the variance, where is possible to see the latent variable 1 separated the LC, OO, and OA groups with respect to the OC group (Figure 2). The second principal component (PC-2) explains 20% of the variance. Variations in these distributions can be explained, in part, by changes in the bile acid profile derived from the diet. These changes can be observed more clearly in (Figure 3), where PC-1 explains 42% of the variance, and the variable latent 1 completely separates the OO and OA groups in relation to the OC group, while the latent variable 2 further separates the two groups fed the supplemented diets along PC-2.

**Figure 4:**
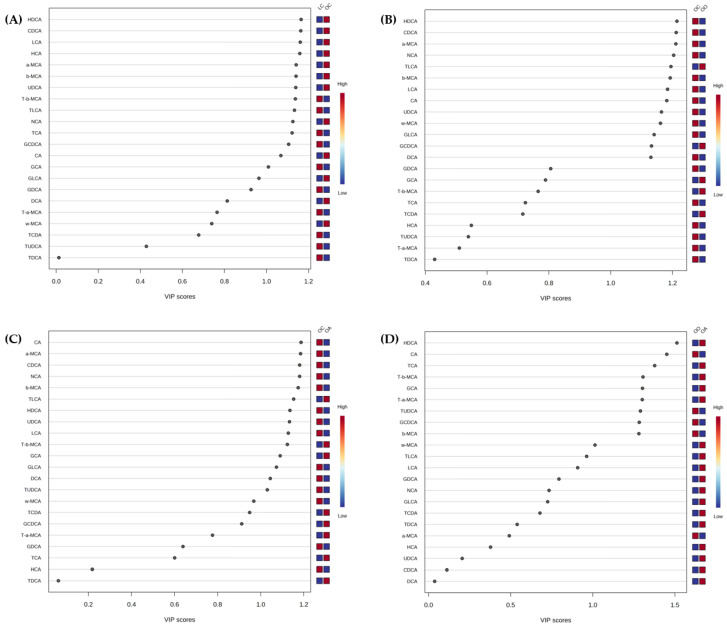
Variable importance in the projection (VIP) scores plots are shown for (**A**) LC and OC; (**B**) OC and OO; (**C**) OC and OA; and (**D**) OO and OA. VIP score (*p* < 0.05) of bile acids identified according to PLS-DA for each diet at the end of the experimental trial (Week 8) generated using MetaboAnalyst 5.0 software after data processing. The coloured boxes on the right indicate the relative concentrations of each bile acid in each group and are organised by descending order of VIP score. Red colour indicates high, and blue colour indicates low. LC, lean control: lean Zucker rats fed a standard diet; OC, obese control: obese Zucker rats fed a standard diet; OO, obese onion 10%: obese Zucker rats fed a standard diet containing 10% onion; OA, obese apple 10%: obese Zucker rats fed a standard diet containing 10% apple. CA, cholic acid; CDCA, chenodeoxycholic acid; DCA, deoxycholic acid; GCA, glycocholic acid; GCDCA, glycochenodeoxycholic acid; GDCA, glycodeoxycholic acid; GLCA, glycolithocholic acid; HCA, hyocholic acid; HDCA, hyodeoxycholic acid; LCA, lithocholic acid; a-MCA, α-muricholic acid; b-MCA, β-muricholic acid; w-MCA, ω-muricholic acid; NCA, nutriacholic acid; TCA, taurocholic acid; TCDA, taurochenodeoxycholic acid; TDCA, taurodeoxycholic acid; TLCA, taurolithocholic acid; T-a-MCA, tauro-α-muricholic acid; T-b-MCA, tauro-β-muricholic acid; TUDCA, tauroursodeoxycholic acid; UDCA, ursodeoxycholic acid.

To analyse the metabolites playing a more significant role, four different comparisons—LC vs. OC; OO vs. OC; OA vs. OC; and OO vs. OA (Figure 4A–D)—were evaluated. A PLS-DA of the bile acid profiles at Week 8 was carried out to better determine the differences among the diet consumption. The variable importance in the projection (VIP) scores obtained from the loading coefficients of the PLS-DA model indicated the metabolites that contributed to the observed group separation.

**Figure 5:**
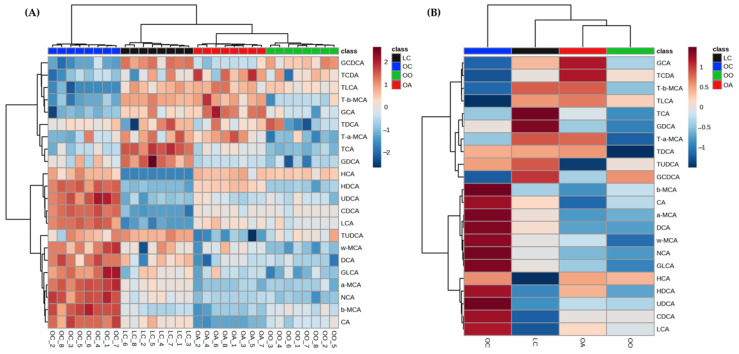
Heatmap of the individual (**A**) and average (**B**) faecal bile acid relative concentration in each group of the study at the end of the experimental trial (Week 8) generated using MetaboAnalyst 5.0 software after data processing. LC, lean control: lean Zucker rats fed a standard diet; OC, obese control: obese Zucker rats fed a standard diet; OO, obese onion 10%: obese Zucker rats fed a standard diet containing 10% onion; OA, obese apple 10%: obese Zucker rats fed a standard diet containing 10% apple. CA, cholic acid; CDCA, chenodeoxycholic acid; DCA, deoxycholic acid; GCA, glycocholic acid; GCDCA, glycochenodeoxycholic acid; GDCA, glycodeoxycholic acid; GLCA, glycolithocholic acid; HCA, hyocholic acid; HDCA, hyodeoxycholic acid; LCA, lithocholic acid; a-MCA, α-muricholic acid; b-MCA, β-muricholic acid; w-MCA, ω-muricholic acid; NCA, nutriacholic acid; TCA, taurocholic acid; TCDA, taurochenodeoxycholic acid; TDCA, taurodeoxycholic acid; TLCA, taurolithocholic acid; T-a-MCA, tauro-α-muricholic acid; T-b-MCA, tauro-β-muricholic acid; TUDCA, tauroursodeoxycholic acid; UDCA, ursodeoxycholic acid.

The BAs of faecal samples showed significant changes, and the percentage of change of each feature has been calculated by the difference between the abundances for both samples and then dividing this difference by the abundance for the control group and is expressed as a percentage. An upward arrow to indicate an increase in percentage and a downward arrow to indicate a decrease in percentage were used (Table 4). In order to provide an intuitive visualization and see how the 22 BAs changed across samples in the different study groups, a hierarchical clustering heatmap was performed using standardised values, a Pearson distance measure, and a Ward clustering algorithm (Figure 5). The colour shows the relative concentration of each bile acid among the different samples. A red colour means that a sample, for a given bile acid, has a read count that is about 2 standard deviations greater than the mean count of that bile acid in all samples, while the blue colour represents the opposite.

The sample clustering shows four clear clusters, corresponding to each group of diet (LC, OC, OA, and OO). Regarding variable clustering, the primary and secondary BAs are clearly influenced by the OC group, while the glycoconjugated and tauroconjugated BAs seemed to be more related to the supplemented diets (OO and OA).

Certain differences were observed, in the case of GCA, and some of the tauroconjugated (TLCA, TCDA, TDCA, T-α-MCA, and T-β-MCA) have relatively high concentrations, particularly in the OA group with respect to the OO group, where T-α-MCA, T-β-MCA, and mainly TDCA have a relatively low concentration in the OO group.

The rest of the BAs, in general, had a relatively low concentration in comparison with the obese control (OC) group. On the other hand, TUDCA exhibited a low concentration in the OA group, while the opposite was observed in the groups that consumed the standard diet (LC and OC). HCA showed a relatively low concentration in the LC group compared to the rest of the groups.

## 4. Discussion

In the present study, as expected, obese Zucker rats were significantly hyperphagic compared to the lean controls, resulting in a greater food intake and body weight [33]. However, when the food efficiency ratio was calculated in the OO and OA groups, it was significantly lower in comparison to the OC group, suggesting a positive effect of onion and apple dietary interventions. Similar results have been reported by Cho et al. (2013) [42] and Yoshinari et al. (2012) [43], confirming the body-weight-reducing effect of apples and onions, respectively.

Regarding the prebiotic effect and the impact in the short-chain fatty acid composition and bile acid content of the dietary interventions, obese rats showed disturbances in the gut microbiota and the levels of SFCAs and BAs. However, some of these changes have been restored to normal levels after dietary supplementation with onion and apple. The benefits of onion and apple feeding on animals have been reported [33,34,44,45].

The link between the gut microbial composition and/or obesity-related pathologies has been and will continue to be investigated by different research groups [46,47,48]. In this context, it is common to use rodents as a model study; for example, the colonization of germ-free mice with microbiota harvested from conventionally raised mice leads to an increase in body fat content and insulin resistance [49]. Another study in genetically obese rats carrying a defective leptin receptor (Zucker fa/fa) suggested that *Bifidobacterium* spp. may have a role in defining an obese or lean phenotype [50]. In the present study, at the beginning of the experiment, *Enterobacter* spp. did not differ between the lean and obese groups. The total aerobic bacteria count, *Escherichia coli*, and total coliform bacteria count reflected higher levels in the obese rats compared with the lean rats, as opposed to *Clostridium* spp., *Bifidobacterium* spp., and *Lactobacillus* spp. that showed lower levels in the obese rats compared with the lean rats. The effect of a high-fat diet on gut microbiota variations has been extensively investigated, indicating a possible role in the development of obesity [51]. In addition, some studies have investigated the benefit of onion and apple feeding in order to assess the effect of polyphenols on the modulation of intestinal microbiota [34,35,38,52]. In the current study, although in all obese groups, at the beginning of the study, the total aerobic bacteria and *Escherichia coli* levels were higher in comparison with the LC group, the onion/apple feeding for 8 weeks caused a significant reduction in both. In this sense, it is important to take into consideration that it has been demonstrated that increased levels of Gram-negative bacteria, which could include *E. coli*, could be related to high plasma endotoxin concentrations and inflammatory markers associated with obesity, as evidenced in animal models [53,54] and humans [55,56]. In addition, although at the start of the feeding, *Enterobacter* spp. did not differ between the lean and obese groups, after 8 weeks of experimental feeding, OC rats showed higher levels in comparison with LC rats, and the OO and OA groups showed reduced levels compared with the OC group. Our results are consistent with previous studies that have reported an increase in Enterobacteriales in diet-induced obese rats [57]. Another study focused on specific species, such as *Enterobacter cloacae*, in germfree mice induce obesity and insulin resistance [58]. Moreover, after administration of polyphenol resveratrol in a dextran sulfate sodium-induced colitis rat model, increased *Bifidobacterium* and *Lactobacillus* counts associated with a decrease in enterobacteria proliferation [59] were shown. In addition, O’Connor et al. (2019) [60] reported that ingestion of components from cranberry modulates the microbiota in a manner that may be beneficial by enriching *Bacteroidaceae* and reducing *Enterobacteriaceae* levels in the gut microbiota. Diet is one of the factors that can positively modulate intestinal microbiota composition. Particularly, polyphenols may exert anti-inflammatory, antioxidant, anticancer, and antidiabetic activities by positively modulating the gut microbiota. Polyphenols have been shown to positively modulate the bacterial component, increasing *Lactiplantibacillus* spp. and *Bifidobacterium* spp. involved in the protection of the intestinal barrier [61]. Hsu et al. (2020) [62] reported that perinatal resveratrol therapy in female Sprague–Dawley rats increased the abundance of *Lactobacillus* and *Bifidobacterium* in male adult offspring. The intestinal microbiota plays a fundamental role in improving their absorption, as intestinal bacteria seem to maintain a reciprocal relationship with them, increasing their bioavailability.

Short-chain fatty acids (SCFAs), which mainly include acetate, propionate, and butyrate, are the major metabolic products of fermentation by colonic intestinal bacteria through dietary fibre in the intestine and some amino acids [63]. Therefore, the presence of dietary fibre in the lumen reflects the activity of the bacterial flora, and SCFA receptors possibly monitor the bacteria for host defence [64]. Some studies suggest that both acetic acid and butyric acid are capable of binding to the anti-inflammatory receptor GPR43, greatly inhibiting the inflammatory response [65,66]. The evidence suggests that increased dietary fibre consumption can positively influence metabolic health by altering gut microbiota. The increased abundance of fermenting species may increase the production and availability of SCFAs, which, in turn, can reduce hyperlipidaemia, hyperglycaemia, hyperinsulinemia, and hypercholesterolemia in a wide range of cohorts, both healthy and metabolically challenged [67]. In this line, improved lipid profile and glucose metabolism, reduced biomarkers of hepatic injury, improved oxidative stress, inflammatory and vascular injury biomarkers, metabolic hormones, endothelial function, and a decreased proinflammatory gene expression of NLRP3, NFKβ1, and COX2 have been previously reported [33]. The microbial activity in the large intestine is generally increased by diets containing a high soluble fibre content. In the present study, the positive effects on the increase in the concentration of some SCFAs, such as acetic, propionic, isobutyric, and valeric acids in the obese groups that consumed the diet enriched with onion (OO) and apple (OA) compared to the obese control (OC) group, indicate the stimulation of the microbiota was probably caused by the fibre present in the onion and apple. In the same line as our findings, this effect has also been observed in other studies, which show an increase in microbial activity resulting in a higher production of SCFAs with a diet rich in apple fibre. Sembries et al. (2006) [68] reported a significant increase in acetic acid in the caecal content of rats fed with apple extraction juices for 4 weeks compared to the control group. At the same time, Aprikian et al. (2001) [69] reported a significant increase in butyrate in the caecal content of rats compared to controls, after the ingestion of freeze-dried pectin. Ravn-Haren et al. (2018) [70] showed a tendency toward an increased concentration of caecal acetate and significantly increased caecal butyrate concentration in rats fed with apple pomace for 4 weeks. Furthermore, Roldán-Marín et al. (2009) [39] showed a significant increase in the formation of caecal propionate and butyrate in rats fed different onion by-products for 4 weeks. In addition, Martinez et al. (2023) [71] showed that the intake of sorghum flour increased the propionic acid in male Wistar rats fed with a high-fat high-fructose diet and modulated the gut microbiota composition and the abundance of SCFA-producing bacteria. On the other hand, in the present study, it should be mentioned that low concentrations of caproic acid were also observed, without any significant difference between the groups, as a result of bacterial degradation of certain amino acids.

Although mouse models have long been used to study essential aspects of bile acid metabolism, compared with humans, there is a clear species difference in the composition of BAs [72]. Human primary BAs are cholic acid (CA) and chenodeoxycholic acid (CDCA). While CDCA is abundant in human bile, it is not in mice because CDCA is converted to muricholic acid (MCA); therefore, the primary BAs in rodents are α- and β-muricholic acids (α-MCA, β-MCA). Previous studies suggest that despite UDCA being a very minor constituent in the total bile acid pool in mice, it could be also an apparent biosynthetic precursor to β-MCA by a mechanism that remains unclear [73]. Therefore, these differences are an essential consideration when trying to extrapolate the findings in mouse BAs to humans. Interestingly, in the present study, CDCA and some of the secondary BAs (HCA and UDCA) resulted in decreased excretion in the LC group in comparison with the OC group. The diet enriched with onion and apple also resulted in a decreased excretion of some BAs, mainly of the primary (CA, α-MCA, and β-MCA) and secondary type (ω-MCA, HDCA, NCA, DCA, and LCA). Some studies in animal models suggest that the higher fat content of an animal-based diet increases bile secretion, augmenting the concentration of hydrophobic BAs in the intestine. Mainly CDCA leads to a decrease in *Lactobacillus* and an increase in *Clostridium* subcluster XIVa, which generates a persistent degree of chronic inflammation and tumour progression [74]. On the other hand, many studies have generally shown the negative impact of BAs on the integrity of the membrane of intestinal bacteria due to a detergent effect, which causes a decrease in the production of membrane proteins and oxidative/nitrosative stress [75]. This triggers an increase in permeability and cell death [76], as Gram-negative bacteria is more resistant to BAs than Gram-positive bacteria [77]. Consistent with these studies, Balderas et al. (2022) [33] found metabolic benefits accompanied by the systemic improvement of oxidative stress, inflammation, and vascular injury biomarkers in rats fed onion- and apple-enriched diets compared with obese control rats. It is important to highlight that, in the present study, obese rats have shown higher levels of primary and secondary BAs and lower levels of *Lactibacillus* spp. It was reported that the proportion of CDCA and CA in faeces was associated with the colon transit [78]. When the hepatic BA synthesis increases and the colon is exposed to more BAs, they will promote fluid and electrolyte secretion. These alterations may explain the mechanisms underlying some cases of BA diarrhoea [79]. The alteration to the gut microbiota ecosystem is characterised by a reduced amount of probiotic bacteria and is primarily determined by intrinsic and extrinsic factors, which can give rise to diseases not only in the gastrointestinal tract but also at a systemic level.

In general, BAs (including DCA, LCA, NCA, HDCA, and ω-MCA) were increased in the OC group; all of them are secondary BAs. It is well known that the secondary BAs are derived from intestinal microbial biotransforming; in this context, different *Clostridium* species have been identified for their ability to transform primary BAs into secondary BAs in obesity models due to dysbiosis of the gut microbiota [80]. In this respect, our results in terms of the *Clostridium* spp. levels are closely aligned with this existing knowledge. Generally, the hydrophobic bile acids LCA and DCA are cytotoxic, and the hydrophilic bile acid UDCA and its derivative tauroursodeoxycholic acid (TUDCA) are cytoprotective [81]. The more hydrophobic the BAs are, only a small amount will be reabsorbed back into the enterohepatic circulation. Consequently, the amount of that BA in the faeces is higher. In previous studies, some secondary BAs, especially DCA, when they are present in high levels in the enterohepatic circulation, are associated with the contributions of colorectal cancer, gallstones, cirrhosis, and other gastrointestinal diseases [82,83,84]. Such is the case in a study by Yoshimoto et al. (2013) [84] that showed that the gut bacterial metabolite DCA promoted the development of obesity-associated hepatocellular carcinoma, which was induced with 7,12-dimethylbenzanthracene (DMBA) in a mouse model. In humans, high faecal excretion of DCA and LCA has been previously linked with the incidence of colorectal cancer after a high-fat diet intake [85]. Animal experiments also revealed that the secondary BAs promote tumorigenesis compared to the primary BAs. Therefore, the relation between secondary BAs/primary BAs, for example, the DCA/CA ratio, helps determine cancer risk [86]. It has been demonstrated that the production of DCA is via the catalysis of the 7α-dehydroxylase that is present in bacteria, including species of *Eubacterium* and *Clostridium* genus. Therefore, the observed increase in faecal DCA in OC rats could be related to the enhanced population of 7α-dehydroxylase containing bacteria [6].

In the liver, secondary BAs are also conjugated with glycine and taurine, producing GCA, GDCA, GCDCA, GLCA, TCA, TUDCA, TCDA, TDCA, TLCA, T-α-MCA, and T-β-MCA. In the present study, the levels of conjugated BAs in faeces were decreased in the OC group, specifically, the levels of TUDCA, T-α-MCA, and GLCA. The conjugated BAs act as an emulsifier, aiding the absorption of ingested fat, which explains the reduced levels of conjugated BAs in faeces. In addition, the decreasing levels of conjugated BAs are contributed by gut microbiota that is capable of the hydrolysis of BAs, producing a range of secondary BAs [87,88]. Zheng et al. (2017) [89] found a correlative relationship between BAs and the high-fat diet-induced alteration of gut microbiota in obese mice with increased concentrations of conjugated BAs TCA, TCDCA, T-α-MCA, T-β-MCA, GCA, and GCDCA. In the present study, onion and apple diets had considerable effects on the concentration of both conjugated and unconjugated bile acids by increasing, in general, the amount of taurine- and glycine-conjugated BAs and decreasing the concentration mainly of the primary and secondary BAs. Consistent with the findings in the present study, González-Peña et al. (2017) [34] previously reported an increase in CA, MCA, CDCA, and UDCA concentrations in rats fed a high-cholesterol diet significantly prevented in rats fed the high-cholesterol enriched with onion diet. In addition, rats fed the high-cholesterol enriched with onion diet showed a tendency to augment the concentration of conjugated forms compared with the rats fed a high-cholesterol diet. Kumari and Augusti (2007) [90] studied the lipid-lowering action of *S*-methyl cysteine sulfoxide isolated from *Allium cepa* Linn in Sprague–Dawley rats fed a 1% cholesterol diet, reporting an increase in the excretion of BAs and sterols in rats treated with *S*-methyl cysteine sulfoxide attributed to a reduction in endogenous lipogenesis, an increased catabolism of lipids, and the subsequent excretion of metabolic by-products through the intestinal tract. In the same line, Hall et al. (2020) [91] reported an increased tauroursodeoxycholate in cats after consuming apple pomace compared with short-chain fructooligosaccharides, and Hosoyamada and Yamada (2017) [92] showed and increased excretion of BAs into the faeces of rats fed apple polyphenol, fish oil, or fish oil and apple polyphenol for 4 weeks, suggesting the beneficial effect of polyphenols.

## 5. Conclusions

This study provides new insights that onion and apple intervention has positive effects in obese Zucker (fa/fa) rats through a prebiotic effect, an increase in the concentration of some SCFAs, such as acetic, propionic, isobutyric, and valeric acids, a decrease in the excretion of some BAs, mainly of the primary (CA, α-MCA, and β-MCA) and secondary type (ω-MCA, HDCA, NCA, DCA, and LCA), and an increase in conjugated BAs by enhancing, in general, the amount of taurine- and glycine-conjugated BAs. Future research is needed into the contribution of specific bioactive compounds and their metabolites in the regulation of microbiome and the pathways linked to SCFA and BA formation and their relationship with some diseases. Solid insights into these underlying mechanisms to pave the way for preventive strategies targeting metabolites and regulation pathways are needed.

## Figures and Tables

**Table 1:** Prebiotic effect parameters in rats fed the lean control/obese control, obese onion 10%, and obese apple 10% diets for 8 weeks.

	LC	OC	OO	OA
**Caecum**				
pH	7.16 ± 0.39 ^a^	7.22 ± 0.4 ^a^	7.34 ± 0.42 ^a^	7.01 ± 0.29 ^a^
Weight (g fw)	0.55 ± 0.03 ^a^	0.55 ± 0.03 ^a^	0.54 ± 0.02 ^a^	0.54 ± 0.03 ^a^
ABTS^•+^ (μmol TE/100 g fw)	3.16 ± 0.06 ^b^	2.45 ± 0.15 ^a^	3.23 ± 0.13 ^b^	3.00 ± 0.17 ^b^
FRAP (μmol TE/100 g fw)	2.31 ± 0.06 ^b^	1.89 ± 0.05 ^a^	2.30 ± 0.05 ^b^	2.31 ± 0.06 ^b^
**Faeces**				
pH—Week 0	7.00 ± 0.57 ^aA^	6.63 ± 0.39 ^aA^	6.73 ± 0.41 ^aA^	6.71 ± 0.28 ^aA^
pH—Week 8	7.07 ± 0.39 ^abA^	6.73 ± 0.28 ^aA^	7.35 ± 0.43 ^bB^	7.07 ± 0.37 ^abB^
Weight—Week 0 (g fw)	3.19 ± 0.24 ^aA^	3.89 ± 0.30 ^bB^	4.45 ± 0.24 ^cB^	4.30 ± 0.51 ^bcB^
Weight—Week 8 (g fw)	3.02 ± 0.20 ^aA^	3.36 ± 0.34 ^aA^	3.79 ± 0.32 ^bA^	3.35 ± 0.33 ^aA^
Total aerobic bacteria count (log CFU/g fw)—Week 0	8.76 ± 0.07 ^aB^	8.92 ± 0.10 ^bB^	8.97 ± 0.08 ^bB^	8.98 ± 0.10 ^bB^
Total aerobic bacteria count (log CFU/g fw)—Week 8	6.91 ± 0.42 ^aA^	8.79 ± 0.02 ^cA^	8.12 ± 0.06 ^bA^	7.88 ± 0.28 ^bA^
*Enterobacter* spp. (log CFU/g fw)—Week 0	8.16 ± 0.01 ^aB^	8.10 ± 0.14 ^aA^	8.12 ± 0.68 ^aA^	8.08 ± 0.78 ^aB^
*Enterobacter* spp. (log CFU/g fw)—Week 8	7.48 ± 0.16 ^abA^	8.06 ± 0.47 ^cA^	7.84 ± 0.13 ^bcA^	7.20 ± 0.60 ^aA^
β-glucuronidase-positive *Escherichia coli* (log CFU/g fw)—Week 0	8.08 ± 0.65 ^aB^	8.60 ± 0.41 ^bB^	8.77 ± 0.10 ^bB^	8.78 ± 0.18 ^bA^
β-glucuronidase-positive *Escherichia coli* (log CFU/g fw)—Week 8	7.52 ± 0.20 ^aA^	7.99 ± 0.47 ^bA^	8.44 ± 0.18 ^cA^	8.61 ± 0.15 ^cA^
Total coliform bacteria count (log CFU/g fw)—Week 0	8.08 ± 0.65 ^aB^	8.60 ± 0.41 ^bA^	8.77 ± 0.10 ^bB^	8.78 ± 0.18 ^bA^
Total coliform bacteria count (log CFU/g fw)—Week 8	7.52 ± 0.20 ^aA^	7.99 ± 0.47 ^bA^	8.44 ± 0.18 ^cA^	8.61 ± 0.15 ^cA^
Sulfite-reducing *Clostridium* spp. (log CFU/g fw)—Week 0	6.64 ± 0.14 ^bB^	4.57 ± 0.39 ^aA^	4.55 ± 0.54 ^aA^	4.65 ± 0.53 ^aA^
Sulfite-reducing *Clostridium* spp. (log CFU/g fw)—Week 8	6.23 ± 0.02 ^aA^	6.61 ± 0.05 ^bB^	6.42 ± 0.09 ^abB^	6.57 ± 0.33 ^bB^
*Bifidobacterium* spp. (log CFU/g fw)—Week 0	9.09 ± 0.03 ^bB^	8.49 ± 0.13 ^aB^	8.50 ± 0.40 ^aB^	8.51 ± 0.35 ^a*B*^
*Bifidobacterium* spp. (log CFU/g fw)—Week 8	8.32 ± 0.11 ^cA^	7.88 ± 0.04 ^abA^	7.77 ± 0.14 ^aA^	7.99 ± 0.03 ^b*A*^
*Lactobacillus* spp. (log CFU/g fw)—Week 0	9.07 ± 0.09 ^bB^	8.58 ± 0.13 ^aB^	8.53 ± 0.24 ^aB^	8.55 ± 0.06 ^aB^
*Lactobacillus* spp. (log CFU/g fw)—Week 8	8.35 ± 0.10 ^dA^	7.67 ± 0.02 ^a*A*^	7.85 ± 0.08 ^bA^	8.11 ± 0.02 ^cA^

Data are presented as mean ± SD (*n* = 8/group). LC, lean control: lean Zucker rats fed a standard diet; OC, obese control: obese Zucker rats fed a standard diet; OO, obese onion 10%: obese Zucker rats fed a standard diet containing 10% onion; OA, obese apple 10%: obese Zucker rats fed a standard diet containing 10% apple. Mean values within a row with different superscript small letters were significantly different; *p* < 0.05 (one-way ANOVA and posterior Tamhane’s T2 and Bonferroni post hoc tests were used as appropriate; superscript small letters indicate Tamhane’s T2 post hoc test). Mean values within a column in the same group (LC, OC, OO, and OA) for the same parameter (in faeces) with different superscript capital letters were significantly different; *p* < 0.05 (Student’s *t*-test). Superscript capital letters indicate that equal variances were not assumed.

**Table 2:** Short-chain fatty acid concentration in faeces from rats fed lean control/obese control, obese onion 10%, and obese apple 10% diets for 8 weeks.

	LC	OC	OO	OA
Acetic acid (µmol/g fw)	46.32 ± 4.54 ^b^	35.58 ± 1.45 ^a^	55.01 ± 4.98 ^c^	45.42 ± 3.92 ^b^
Propionic acid (µmol/g fw)	5.47 ± 0.75 ^c^	2.61 ± 0.03 ^a^	4.40 ± 0.17 ^b^	4.42 ± 1.02 ^b^
Isobutyric acid (µmol/g fw)	0.46 ± 0.06 ^c^	0.32 ± 0.01 ^a^	0.39 ± 0.04 ^b^	0.35 ± 0.02 ^ab^
Butyric acid (µmol/g fw)	0.27 ± 0.02 ^c^	0.13 ± 0.02 ^b^	0.10 ± 0.02 ^a^	0.13 ± 0.01 ^b^
Isovaleric acid (µmol/g fw)	0.31 ± 0.05 ^c^	0.19 ± 0.05 ^b^	0.19 ± 0.04 ^b^	0.12 ± 0.02 ^a^
Valeric acid (µmol/g fw)	0.11 ± 0.01 ^a^	0.15 ± 0.02 ^b^	0.24 ± 0.01 ^c^	0.37 ± 0.02 ^d^
Caproic acid (µmol/g fw)	0.13 ± 0.01 ^b^	0.12 ± 0.004 ^ab^	0.12 ± 0.01 ^ab^	0.11 ± 0.01 ^a^

Data are presented as mean ± SD (*n* = 8/group). LC, lean control: lean Zucker rats fed a standard diet; OC, obese control: obese Zucker rats fed a standard diet; OO, obese onion 10%: obese Zucker rats fed a standard diet containing 10% onion; OA, obese apple 10%: obese Zucker rats fed a standard diet containing 10% apple. Mean values within a row with different superscript small letters were significantly different; *p* < 0.05 (one-way ANOVA and posterior Tamhane’s T2 and Bonferroni post hoc tests were used as appropriate, and small letters indicate Tamhane’s T2 post hoc test).

**Table 3:** Bile acid detection using HPLC-QTOF MS from faecal samples in rats fed the lean control/obese control, obese onion 10%, and obese apple 10% diets for 8 weeks.

Bile Acid (Abbreviation)	Formula	RT (min)	Monoisotopic Mass	(M-H)	Fragments
Tauro-α-muricholic acid (T-α-MCA)	C_26_H_45_NO_7_S	2.88	515.2917	514.284398	514.2897; 496.3058; 479.3058; 358.7687; 80.9621; 65.6705
Tauroursodeoxycholic acid (TUDCA)	C_26_H_45_NO_6_S	3.27	499.2968	498.289483	498.2880; 479.9107; 465.9335; 393.2521; 159.0796; 96.9606
Tauro-β-muricholic acid (T-β-MCA)	C_26_H_45_NO_7_S	3.29	515.2917	514.284398	514.2897; 496.3058; 479.3058; 358.7687; 80.9621; 65.6705
ω-muricholic acid (ω-MCA)	C_24_H_40_O_5_	3.35	408.2876	407.280298	407.2799; 391.2347; 373.1968; 345.2663; 179.0411; 59.011
Taurocholic acid (TCA)	C_26_H_45_NO_7_S	3.37	515.2917	514.284398	514.2897; 496.9256; 479.3105; 357.2196; 80.9621
Glycocholic acid (GCA)	C_26_H_43_NO_6_	3.40	465.3090	464.301762	464.3091; 447.1784; 405.2479; 379.2552; 357.1239
α-Muricholic acid (α-MCA)	C_24_H_40_O_5_	4.00	408.2876	407.280298	407.2791; 389.2682; 373.2247; 345.2770; 60.0348
β-muricholic acid (β-MCA)	C_24_H_40_O_5_	4.17	408.2876	407.280298	407.2805; 389.2735
Taurochenodeoxycholic acid (TCDA)	C_26_H_45_NO_6_S	4.35	499.2968	498.289483	498.2881; 480.2670; 466.2540; 388.2578; 374.1424; 80.9626
Hyocholic acid (HCA)	C_24_H_40_O_5_	4.70	408.2876	407.280298	407.2805; 389.2735
Ursodeoxycholic acid (UDCA)	C_24_H_40_O_4_	4.85	392.2927	391.285384	391.2526; 373.9815; 357.7511; 329.7645; 221.0703; 59.0103
Glycodeoxycholic acid (GDCA)	C_26_H_43_NO_5_	4.93	449.3141	448.306847	448.3165; 433.2967; 407.2899; 389.2625; 329.0834; 74.0232
Glycochenodeoxycholic acid (GCDCA)	C_26_H_43_NO_5_	5.16	449.3141	448.306847	448.3165; 433.2967; 407.2899; 389.2625; 329.0834; 74.0232
Taurodeoxycholic acid (TDCA)	C_26_H_45_NO_6_S	5.33	499.2968	498.289483	498.2881; 480.2670; 466.2540; 388.2578; 374.1424; 80.9626
Cholic acid (CA)	C_24_H_40_O_5_	5.53	408.2876	407.280298	407.2808; 391.2378; 371.2674; 345.2730; 289.7773; 131.6716
Hyodeoxycholic acid (HDCA)	C_24_H_40_O_4_	5.65	392.2927	391.285384	391.2839; 373.2686; 345.2772; 327.2668; 284.2067; 59.0152
Nutriacholic acid (NCA)	C_24_H_38_O_4_	6.65	390.2770	389.269734	
Taurolithocholic acid (TLCA)	C_26_H_45_NO_5_S	6.75	483.3018	482.294569	482.2910; 464.4037; 389.2734; 349.1847; 79.9556
Deoxycholic acid (DCA)	C_24_H_40_O_4_	7.66	392.2927	391.285384	391.2905; 355.2557; 345.2856; 327.2615; 140.6033; 57.0309
Chenodeoxycholic acid (CDCA)	C_24_H_40_O_4_	9.22	392.2927	391.285384	391.2872; 373.9914; 345.2780; 329.2756; 140.6033; 59.0132
Lithocholic acid (LCA)	C_24_H_40_O_3_	16.33	376.2977	375.290469	375.2906; 357.2796; 329.3094; 191.4657; 50.0429
Glycolithocholic acid (GLCA)	C_26_H_43_NO_4_	22.69	433.3192	432.311933	433.3379; 405.2467; 387.2367; 373.2224; 359.1364; 59.0162

**Table 4:** Bile acid differences from faecal samples in rats fed the lean control/obese control, obese onion 10%, and obese apple 10% diets for 8 weeks.

					Week 0	Week 8			
Type	Abbreviation	Formula	RT (min)	Monoisotopic Mass	% Change Lean vs. Obese	% Change LC vs. OC	% Change OO vs. OC	% Change OA vs. OC	% Change OO vs. OA
Primary	CA	C_24_H_40_O_5_	5.53	408.2876	↑	↓	↓	↓	↑
Primary	CDCA	C_24_H_40_O_4_	9.22	392.2927	↓	↓	↓	↓	↓
Primary (mouse)	α-MCA	C_24_H_40_O_5_	4.00	408.2876	↓	↓	↓	↓	↑
Primary (mouse)	β-MCA	C_24_H_40_O_5_	4.17	408.2876	↓	↓	↓	↓	↑
Secondary (mouse)	ω-MCA	C_24_H_40_O_5_	3.35	408.2876	↑	↓	↓	↓	↓
Secondary	HCA	C_24_H_40_O_5_	4.70	408.2876	↓	↓	↓	↓	↓
Secondary	UDCA	C_24_H_40_O_4_	4.85	392.2927	↓	↓	↓	↓	↓
Secondary	HDCA	C_24_H_40_O_4_	5.65	392.2927	↓	↓	↓	↓	↓
Secondary	NCA	C_24_H_38_O_4_	6.65	390.2770	↓	↓	↓	↓	↓
Secondary	DCA	C_24_H_40_O_4_	7.66	392.2927	↓	↓	↓	↓	↓
Secondary	LCA	C_24_H_40_O_3_	16.33	376.2977	↓	↓	↓	↓	↓
Glycoconjugated	GCA	C_26_H_43_NO_6_	3.40	465.3090	↑	↑	↑	↑	↓
Glycoconjugated	GDCA	C_26_H_43_NO_5_	4.93	449.3141	↑	↑	↓	↓	↓
Glycoconjugated	GCDCA	C_26_H_43_NO_5_	5.16	449.3141	↑	↑	↑	↑	↑
Glycoconjugated	GLCA	C_26_H_43_NO_4_	22.69	433.3192	↓	↓	↓	↓	↓
Tauroconjugated	TUDCA	C_26_H_45_NO_6_S	3.27	499.2968	↑	↑	↓	↓	↑
Tauroconjugated	TCA	C_26_H_45_NO_7_S	3.37	515.2917	↑	↑	↓	↑	↓
Tauroconjugated	TCDA	C_26_H_45_NO_6_S	4.35	499.2968	↑	↑	↑	↑	↓
Tauroconjugated	TDCA	C_26_H_45_NO_6_S	5.33	499.2968	↑	↑	↓	↑	↓
Tauroconjugated	TLCA	C_26_H_45_NO_5_S	6.75	483.3018	↑	↑	↑	↑	↓
Tauroconjugated (mouse)	T-α-MCA	C_26_H_45_NO_7_S	2.88	515.2917	↑	↑	↓	↑	↓
Tauroconjugated (mouse)	T-β-MCA	C_26_H_45_NO_7_S	3.29	515.2917	↓	↑	↑	↑	↓

## Data Availability

Data are contained within the article.

## Supplementary Materials

The following supporting information can be downloaded at: https://www.mdpi.com/article/10.3390/nu15133035/s1, Supplementary Materials and Methods S2.1. Onion and Apple Powder Preparation, Table S1: Nutritional composition, phytochemical compounds, and antioxidant activity of onion and apple powder; S2.2. Animal Model and Experimental Design, Table S2: Composition of the experimental diets.
