# Supplementary material for: Bile Acids and Short-Chain Fatty Acids Are Modulated after Onion and Apple Consumption in Obese Zucker Rats
Source: Nutrients. 2023 Jul 5;15(13):3035. doi: 10.3390/nu15133035 (PMC10347221; doi:10.3390/nu15133035)
Supplement: Supplementary file 1 [file nutrients-15-03035-s001.zip › nutrients-2472339-supplementary.pdf]

S2. Supplementary Materials and Methods

S2.1. Onion and Apple Powder Preparation

Onions (*Allium cepa* L. var. *cepa*, 'Recas') and apples (*Mallus pumila* Mill., 'Golden delicious') were purchased from a local fresh market in Madrid, Spain. The onions were hand-peeled and cut into 10 mm pieces. The apples were washed, divided into quarters without core, cut into 2 cm pieces with skin and quickly packed. Approximately 120 g of fresh-cut onion and apple were packaged in low gas permeability film bags (BB3255, Cryovac, Barcelona, Spain) and treated by high pressure (400 MPa/25 °C/5 min, and 400 MPa/35 °C/5 min, respectively) (High Pressure Iso-Lab System, model FPG7100:9/2C, Stansted Fluid Power Ltd., Essex, UK). After the high pressure treatment, the onion and apple were frozen with liquid nitrogen, freeze-dried in a lyophilizer (model Lyoalfa, Telstar, S.A., Barcelona, Spain) and pulverized with an ultra-centrifugal mill ZM 200 (Retsch GmbH, Haan, Germany), obtaining a fine powder (final size particle ≤ 250 μm), and stored at −20 ± 0.5 °C until use. Supplementary Table S1 shows nutritional composition, phytochemical compounds and antioxidant activity of the onion and the apple powder. Analyses were carried out using the methods previously cited in Balderas et al. (2022) [33].

Supplementary Table S1. Nutritional composition, phytochemical compounds and antioxidant activity of onion and apple powder [33].

	**Onion Powder**	**Apple Powder**
Protein (g/100 g)	9.87 ± 0.07	1.60 ± 0.01
Lipids (g/100 g)	1.35 ± 0.05	1.45 ± 0.07
Carbohydrates (g/100 g)	79.12 ± 3.18	85.67 ± 3.84
Glucose (g/100 g)	32.81 ± 1.25	15.23 ± 1.81
Fructose (g/100 g)	19.04 ± 2.50	31.37 ± 3.08
Sucrose (g/100 g)	7.74 ± 1.28	5.93 ± 0.85
Total dietary fibre (g/100 g)	16.86 ± 1.23	14.43 ± 1.89
Soluble fibre (g/100 g)	3.71 ± 0.07	3.14 ± 0.56
Insoluble fibre (g/100 g)	13.15 ± 0.97	11.29 ± 1.63
Pectin (g/100 g)	ND	2.67 ± 0.67
Ash (g/100 g)	3.59 ± 0.15	0.98 ± 0.04
Total phenols (mg GAE/100 g)	948.78 ± 39.75	2077.03 ± 74.42
Quercetin (mg/100 g)	1.56 ± 0.56	ND
Quercetin 3-*O*-rutinoside (mg/100 g)	ND	0.33 ± 0.07
Quercetin 3-*O*-arabinoside (mg/100 g)	ND	2.39 ± 0.30
Quercetin 3-*O*-xyloside (mg/100 g)	ND	3.82 ± 0.004
Quercetin 3-*O*-rhamnoside (mg/100 g)	ND	5.87 ± 0.05
Quercetin 3-*O*-glucoside (mg/100 g)	10.62 ± 0.08	2.45 ± 0.60
Quercetin 3-*O*-galactoside (mg/100 g)	ND	5.59 ± 0.79
Quercetin 4′-*O*-glucoside (mg/100 g)	313.53 ± 2.12	ND
Quercetin 3,4′-di-*O*-glucoside (mg/100 g)	1571.16 ± 6.75	7.44 ± 0.36
Quercetin 7,4′-*O*-diglucoside (mg/100 g)	37.95 ± 0.75	ND
Quercetin 3,7,4′-*O*-triglucoside (mg/100 g)	12.29 ± 0.35	ND
Isorhamnetin 3-*O*-glucoside (mg/100 g)	0.45 ± 0.001	ND
Isorhamnetin 4′-glucoside (mg/100 g)	12.95 ± 1.16	ND
Isorhamnetin 3,4′-diglucoside (mg/100 g)	3.18 ± 1.07	ND
Phloridzin (mg/100 g)	18.15 ± 1.57	6.35 ± 0.45
Phloretin (mg/100 g)	ND	0.17 ± 0.01
Phloretin 2′-xyloglucoside (mg/100 g)	ND	3.60 ± 0.14
Epicatechin (mg/100 g)	10.37 ± 0.35	20.81 ± 0.44
Catechin (mg/100 g)	ND	0.41 ± 0.004
Epicatechin dimer 1 (mg/100 g)	ND	4.05 ± 0.04
Epicatechin dimer 2 (mg/100 g)	ND	27.00 ± 0.90
Epigallocatechin (mg/100 g)	ND	11.18 ± 1.29
Procyanidin B2 (mg/100 g)	ND	26.84 ± 7.87
Chlorogenic acid (mg/100 g)	ND	28.15 ± 0.89
Neochlorogenic acid (mg/100 g)	ND	1.36 ± 0.003
Cryptochlorogenic acid (mg/100 g)	ND	1.67 ± 0.13
p-Coumaric acid (mg/100 g)	ND	0.08 ± 0.003
Propionaldehyde (mg/100 g)	15.59 ± 0.89	ND
1-Propanethiol (mg/100 g)	2.50 ± 0.15	ND
Hexanal (mg/100 g)	0.26 ± 0.02	2.17 ± 0.05
2-Methyl 2-pentenal (mg/100 g)	0.47 ± 0.05	ND
Propyl thioacetate (mg/100 g)	0.038 ± 0.0002	ND
Dimethyl trisulphide (mg/100 g)	0.057 ± 0.0004	ND
Dipropyl disulphide (mg/100 g)	2.70 ± 0.37	ND
Methyl propyl trisulphide (mg/100 g)	0.30 ± 0.02	ND
Dipropyl trisulphide (mg/100 g)	1.00 ± 0.09	ND

Ascorbic acid (mg/100 g)	58.75 ± 0.30	51.33 ± 0.80
Total vitamin C (mg/100 g)	71.25 ± 0.20	82.67 ± 0.90
Peroxidase activity (ΔOD/min/100 g)	7.66 ± 1.24	20.74 ± 0.89
Polyphenoloxidase activity (ΔOD/min/100 g)	0.38 ± 0.09	0.91 ± 0.04
$ABTS^{\bullet+}$ (μmol TE/100 g)	1509.45 ± 35.05	1821.79 ± 154.65
$DPPH^{\bullet}$ (μmol TE/100 g)	1129.50 ± 153.22	2589.74 ± 185.31
FRAP (μmol TE/100 g)	1122.55 ± 52.03	1582.95 ± 110.57

Data are presented as mean ± SD (*n* = 3). ND, not determined; GAE, gallic acid equivalents; $ABTS^{\bullet+}$, 2,2′-azinobis(3-ethylbenzothiazoline-6-sulfonic acid) radical cation; $DPPH^{\bullet}$, 2,2-diphenyl-1-picrylhydrazyl radical; FRAP, ferric reducing antioxidant power; TE, trolox equivalents.

S2.2. Animal Model and Experimental Design

The present study was approved by the Spanish Ministry of Economy, Industry and Competitiveness Advisory Committee (project AGL2016-76817-R) and by the Ethics Committee of the Complutense University of Madrid (Spain) (Reference PROEX 133/16). All experiments were performed in compliance with Directive 2010/63/UE regarding the protection of animals used for scientific purposes. All necessary steps were taken to prevent any potential animal suffering. Twenty-four male, homozygous (fa/fa), obese Zucker rats (Crl:ZUC(Orl)-*Lepr*^{fa}) with a body weight of approximately 225 g at the outset, and eight male, heterozygous (fa/+), lean Zucker rats with a body weight of approximately 200 g at the outset, all 7 wk of age, were acquired from Charles River Laboratories, Barcelona, Spain. The animals were housed in groups of four under controlled conditions (12 h light−12 h dark cycle, 22.5 ± 0.5 °C ambient temperature, 50–60% relative humidity). The rats were fed commercial rat pellets (Panlab, SLU, Barcelona, Spain) for 5 days for the adaptation to environmental conditions. The obese Zucker rats were randomly divided into three groups of eight rats each: obese control (OC) group, obese Zucker rats fed a standard diet; obese onion 10% (OO) group, obese Zucker rats fed a standard diet containing 10% onion; and obese apple 10% (OA) group, obese Zucker rats fed a standard diet containing 10% apple. Lean Zucker rats served as a lean control (LC) group: lean Zucker rats fed a standard diet. Three semisynthetic diets were prepared based on the AIN-93M semi-purified rodent diet [36], with comparable levels of gross energy and crude nutrients (Supplementary Table S2). In OO and OA diets, maize starch and cellulose powder were adjusted to compensate for the addition of onion and apple powder, respectively. The doses for onion and apple powder were selected based on the body surface area normalization method [37] and previous studies [38-40], representing an amount of onion and apple that could reasonably be expected to be achieved in the human population having healthy food habits. Water and food were provided ad libitum over the 8-week experimental feeding trial. Body weight and faecal weight were recorded weekly and food intake was recorded daily. The food efficiency ratio and the apparent diet digestibility were calculated according to the formulas published in Balderas et al. (2022) [33].

Supplementary Table S2. Composition of the experimental diets [c] [33].

Component (g/kg)	LC/OC Diet	OO Diet	OA Diet
Onion powder	–	100	–
Apple powder	–	–	100
Casein	200	200	200
Sucrose	100	100	100
Maize starch	470.49	386.14	386.14
Soya oil	50	50	50
Maize oil	80	80	80
Mineral mixture [a]	35	35	35
Vitamin mixture [b]	10	10	10
Cellulose powder	50	34.35	34.35
Choline bitartrate	2.5	2.5	2.5
tert-butylhydroquinone	0.01	0.01	0.01
L-cystine	2	2	2

LC/OC, Lean control/Obese control diet; OO, Obese onion 10% diet; OA, Obese apple 10% diet. [a]Mineral mix for the AIN-93M diet, g/kg (AIN-93M-MX): calcium carbonate anhydrous, 357.00; potassium phosphate monobasic, 250.00; potassium citrate, tripotassium monohydrate, 28.00; sodium chloride, 74.00; potassium sulphate, 46.00; magnesium oxide, 24.00; ferric citrate, 6.06; zinc carbonate, 1.65; sodium meta-silicate 9H_2O, 1.45; manganous carbonate, 0.63; cupric carbonate, 0.30; chromium potassium sulphate 12H_2O, 0.275; boric acid, 0.0815; sodium fluoride, 0.0635; nickel carbonate, 0.0318; lithium chloride, 0.0174; sodium selenate anhydrous, 0.01025; potassium iodate, 0.0100; ammonium paramolybdate 4H_2O, 0.00795; ammonium vanadate, 0.0066; powdered sucrose, 209.806. [b]Vitamin mix for the AIN-93M diet, g/kg (AIN-93M-VX): nicotinic acid, 3.000; calcium pantothenate, 1.600; pyridoxine-HCl, 0.700; thiamin-HCl, 0.600; riboflavin, 0.600; folic acid, 0.200; biotin, 0.200; vitamin B12 (cyanocobalamin) (0.1 % in mannitol), 2.500; vitamin E (all-*rac*-α-tocopheryl acetate, 500 IU/g), 15.000; vitamin A (all-*trans*-retinyl palmitate, 500,000 IU/g), 0.800; vitamin D3 (400,000 IU/g), 0.250; vitamin K1, 0.075; powdered sucrose, 974.655. [c]Diet energy content was calculated using the factors 16.73 kJ/g (4 kcal/g) for protein, 15.69 kJ/g (3.75 kcal/g) for monosaccharides, 16.53 kJ/g (3.95 kcal/g) for disaccharides, 17.49 kJ/g (4.18 kcal/g) for starch, 8.37 kJ/g (2 kcal/g) for dietary fibre, and 37.65 kJ/g (9 kcal/g) for fat. LC/OC diet, 18,540.9 kJ/kg (4431.4 kcal/kg); OO diet, 18,424.8 kJ/kg (4403.6 kcal/kg); OA diet, 18,444.3 kJ/kg (4408.3 kcal/kg).
